# Repeated Head Impacts May Compromise White Matter Integrity and Bimanual Coordination in American Football Players

**DOI:** 10.1177/19417381261494495

**Published:** 2026-10-26

**Authors:** Sebastian D’Amario, Blaire Magee, Allen A. Champagne, Madison E. Wilson, Gabriel Ramírez-García, Nicole S. Coverdale, Douglas J. Cook

**Affiliations:** † Centre for Neuroscience Studies, Queen’s University, Kingston, Ontario, Canada; ‡ Dalhousie University, Halifax, Nova Scotia, Canada; § Duke University, Durham, North Carolina; ‖ Universidad Nacional Autonoma de Mexico, Mexico City, Mexico; ¶ Department of Surgery, Queen’s University, Kingston, Ontario, Canada

**Keywords:** American football, Canada, diffusion tensor imaging, repeated head impacts

## Abstract

**Background:**

Repeated head impacts (RHI) in American Football may contribute to subtle white matter (WM) abnormalities and functional deficits even in the absence of clinically diagnosed concussion.

**Hypothesis:**

Contact athletes would demonstrate tract-specific differences in fractional anisotropy (FA) and mean diffusivity (MD) compared with noncontact controls, and high-exposure (HE) athletes would show greater within-season diffusion change, worse bimanual coordination, and greater symptom burden than low-exposure (LE) athletes.

**Study Design:**

Prospective cohort study.

**Level of Evidence:**

Level 3.

**Methods:**

Twenty-two male varsity American Football players and 13 male noncontact controls underwent preseason diffusion tensor imaging. Football athletes were monitored over a single season using helmet-mounted accelerometers and stratified into HE and LE groups. Tract-specific FA and MD were derived using probabilistic tractography. Pre-post SCAT3 symptom and severity checklist data were collected from all football athletes, and pre-post Kinarm Ball-on-Bar (BOB) bimanual coordination data were available for 12 athletes.

**Results:**

At preseason, contact athletes demonstrated higher FA in 15 of 16 tracts compared with noncontact controls, with lower FA in the forceps minor. MD was lower in 10 of 16 tracts, higher in the bilateral inferior longitudinal fasciculus, and similar in the corticospinal tract and superior thalamic radiation. Over the season, HE athletes sustained more impacts (*P* < 0.001) and greater cumulative *g*-force (*P* = 0.001) than LE athletes. On the BOB task, LE athletes showed modest improvement, whereas HE athletes worsened, yielding a between-group difference in change of 0.59 (95% CI, 0.22-0.96; *P* = 0.005). Generalized estimating equation models accounting for within-athlete tract correlations did not detect HE-versus-LE differences in FA change (Wald χ²(1) = 0.14; *P* = 0.71) or MD change (Wald χ²(1) < 0.01; *P* > 0.99), and SCAT3 symptom and severity scores remained low with no meaningful between-group differences.

**Conclusion:**

In 22 contact athletes, repeated head-impact exposure was associated with preseason WM differences and worsening bimanual coordination across a single season, while longitudinal tract-level DTI and symptom-based clinical measures showed limited between-group change.

**Clinical Relevance:**

Objective motor assessment and advanced neuroimaging may help characterize subtle consequences of RHIs that are missed by routine symptom-based tools.

Head injuries sustained during contact sport participation are recognized increasingly for their potential long-term effects on brain health.^3,7,29^ While concussions have been studied extensively, increasing attention has focused on repeated head impacts (RHI), including impacts that do not produce clinically diagnosed concussion, because they occur much more frequently than concussion. In particular, high school and collegiate American Football players experience an average of 600 to 1400 impacts per season, depending on the level of play and position.^3,8^ A subconcussive impact is defined as a head impact that does not produce observable clinical symptoms yet may still result in neurophysiological changes,^3,29^ making them challenging to detect and address in real time. Nonetheless, neuroimaging studies have highlighted measurable changes in brain structure, particularly in white matter (WM) microstructure. These changes may occur because the biomechanical forces underlying RHI may result in axonal shearing and neuronal disruption, initiating a cascade of neurochemical events that compromise WM integrity.^7,28^

Diffusion tensor imaging (DTI) has been instrumental in quantifying changes in WM integrity with metrics such as fractional anisotropy (FA) and mean diffusivity (MD).^1,39^ FA measures how directionally restricted water movement is, while MD captures the overall amount of diffusion. Alterations in these metrics are associated with microstructural damage processes, including axonal injury and demyelination.^2^ However, findings in RHI-related DTI studies have often been inconsistent, highlighting heterogeneity in WM changes.^11,26,32^ For instance, FA and MD have both been shown to decrease and increase after RHI.^4,5,9,12,21,22,31^ Given these inconsistencies in DTI findings, a multimodal approach may help characterize the structural and functional correlates of RHI.

Given that RHI produces measurable biomechanical strain,^8,14^ it is reasonable to evaluate whether these effects extend to players’ motor behavior. The Kinarm robotic assessment system provides standardized tasks, supported by large normative datasets, that can detect subtle changes in motor performance. In particular, the Ball-on-Bar (BOB) task is a measure of bimanual coordination and has been used to identify clinical consequences in stroke and amyotrophic lateral sclerosis.^27,35^ As people with long-term exposure to RHI frequently report headaches, cognitive slowing, noise sensitivity, short-term memory problems, and disrupted sleep,^20,24,33^ we also included the Sport Concussion Assessment Tool, third edition (SCAT3), symptom and severity checklist to capture subjective symptom burden rather than to diagnose concussion.^19^ Combining these behavioral and clinical measures with neuroimaging provides a more comprehensive view of possible structural and functional correlates of RHI.^31,33,37^

In this study, our aim was to examine WM microstructure in Canadian varsity collegiate American Football players over the course of a football season. By stratifying players into high- and low-exposure groups using helmet accelerometer data, we examined whether cumulative head-impact exposure was associated with motor performance, clinical symptom scores, and within-season diffusion changes. We hypothesized that (1) contact athletes would show tract-specific differences in FA and MD compared with noncontact controls; (2) high-exposure athletes would show a greater magnitude of within-season change in FA and MD than low-exposure athletes; and (3) high-exposure athletes would show greater worsening of bimanual coordination and greater increases in SCAT3 scores across the season than low-exposure athletes. These findings may contribute to a more comprehensive understanding of RHI in contact sports while informing future studies designed to test exposure thresholds and clinical significance.

## Methods

### Participants

A total of 22 male athletes from the Queen’s University Varsity American Football Team (20 ± 1 years old) in Canada, along with 13 noncontact controls (21 ± 2.5 years) participated in this study. Controls were recruited as a convenience sample of young, healthy adult athletes in noncontact sports from the Queen’s University campus during the same study period. Controls were selected to be comparable in age and sex (male) to the American football cohort, and were eligible if they reported no participation in collision and/or contact sports and no diagnosed concussion within the past year. Controls completed 1 magnetic resonance imaging (MRI) session at preseason (PRE) only because the original study design used controls as a cross-sectional baseline comparison for diffusion values in contact athletes entering the monitored season; a second postseason (POST - 30 days after season end) control MRI was not collected. Therefore, controls were not included in longitudinal exposure analyses. American football athletes completed 2 MRI sessions and behavioral assessments at PRE and POST. All participants provided written informed consent, and the study was approved by the Queen’s University Health Sciences Research Ethics Board. American Football participants all played in ≥1 previous season, and were excluded if they had pre-existing injuries, sustained injuries during the season that affected their ability to participate, or had been diagnosed with a concussion within the past year or during the season. Detailed lifetime concussion counts, previous nonfootball head injuries, and other possible sources of RHI were not quantified.

### Impact Monitoring

Head-impact exposure was quantified using *g*-force tracker accelerometers (Artaflex Inc). The devices were mounted inside each athlete’s helmet using 3M Dual Lock Velcro, positioned at the left crown. Each accelerometer, capable of measuring linear acceleration and rotational velocity, was configured with a 15-g threshold to exclude nonrelevant movements (e.g., running, setting helmet down). During games and practices, onsite spotters flagged nonhead impact events (e.g., dropped helmets) for subsequent removal. Flagged events were then reviewed manually and excluded before calculating exposure metrics. No additional custom signal filtering was applied beyond the device trigger threshold and the manufacturer’s internal event-detection processing; analyses were performed using the event-level outputs recorded by the device.

For each athlete, we collected the total impacts and total *g*-force experienced. Total impacts was defined as the count of retained head-impact events across all monitored sessions and total *g*-force was defined as the sum of the device-reported peak linear acceleration values (g) across all retained events. Exposure metrics were computed using an aggregation script that identifies all session-level event files per athlete and sums impact counts and cumulative *g*-force from the same retained events. Athletes were then stratified into high exposure (HE; n = 11) and low exposure (LE; n = 11) groups using a median average frequency of impacts per session of 10 as the threshold.^10^ We selected impact frequency as the primary stratifier because it is a stable, interpretable proxy of cumulative exposure that is less sensitive to per impact measurement noise than single peak kinematic values. We also extracted the total *g*-force (Table 1) as an intensity-weighted summary of seasonal exposure and report it alongside impact counts. We use these exposure metrics primarily for within-cohort stratification and descriptive characterization of relative exposure burden.

**Table 1.** Total season head-impact exposure by group and playing position

Variable	HE	LE	DB (n = 6)	WR (n = 5)	DL (n = 4)	LB (n = 3)	OL (n = 2)	K (n = 1)	RB (n = 1)
Total impacts, N	484 ± 185	222 ± 112	375 ± 247	355 ± 167	430 ± 244	278 ± 192	324 ± 64	33	506
Total *g*-force	16,163 ± 6486	7089 ± 3799	12,367 ± 8510	11,340 ± 5113	13,908 ± 8637	8755 ± 6377	10,632 ± 1576	1086	20,649

Values are mean ± SD for total impacts and total *g*-force for HE, LE, and each position group. DB, defensive back; DL, defensive line; HE, high exposure; K, kicker; LB, linebacker; LE, low exposure; OL, offensive line; RB, running back; TC, training camp; WR, wide receiver.

### MRI Acquisition

All MRI scans were conducted using a Siemens 3.0 T Magnetom Tim Trio system. T1-weighted structural images were acquired using a magnetization prepared rapid gradient echo imaging sequence (repetition time, 1760 ms; echo time, 2.2 ms; inversion time, 900 ms; matrix, 256 × 256; field of view, 256 mm^2^; voxel size, 1 × 1 × 1 mm). We performed spin-echo echo-planar diffusion-weighted imaging with the following parameters: repetition time, 7800 ms; echo time, 95 ms; field of view, 256 mm^2^; matrix, 128 × 128; 60 axial slices; voxel size, 1 × 1 × 1 mm; b-value, 1000 s/mm^2^; and 30 diffusion directions. Three b = 0 reference volumes were collected as nondiffusion-weighted images for distortion correction, motion correction, and tensor estimation. Images were collected at each participant’s scheduled imaging session under consistent conditions. The SCAT3 symptom checklist was self-reported before each MRI session. Only the SCAT3 symptom number and symptom severity scores were analyzed; the full SCAT3 and Maddocks, cognitive, and balance components were not analyzed as outcomes. Higher symptom and severity scores indicate greater symptom burden.

### Neuroimaging Processing

Diffusion data were preprocessed using tools from FMRIB’s Software Library (FSL).^23^ Preprocessing steps included correction for susceptibility-induced distortions using the *topup* tool, correction for eddy currents and motion artifacts using *eddy*, and brain extraction with the *bet* tool. Diffusion tensors were calculated for each voxel using *dtifit* to produce maps of fractional FA and MD metrics for each subject. These maps were aligned to a standardized Montreal Neurological Institute (MNI152 2 mm) template using *FLIRT* and *FNIRT* for linear and nonlinear registration, respectively.

### Tractography

After preprocessing, probabilistic tractography was conducted to analyze WM tracts using BEDPOSTX, which models crossing fibers within each brain voxel.^6^ Streamline density maps, generated using FSL’s *PROBTRACKX* tool, quantified connectivity from the seed region to the target region for each tract. To ensure comparability across subjects, density maps were normalized by dividing each map by its waytotal using FSL’s *fslmaths*. Waytotals represent the total number of streamlines reaching the target mask from the seed mask, accounting for intersubject differences in scan quality or motion. In total, this was done for the following tracts: anterior thalamic radiation, corticospinal tract, forceps major, forceps minor, inferior fronto-occipital fasciculus, inferior longitudinal fasciculus, superior longitudinal fasciculus, superior thalamic radiation, and uncinate fasciculus. Normalized tracts were registered to the MNI152 2-mm standard space using *FLIRT* and *FNIRT* tools. Each tract was thresholded to eliminate low-probability streamlines, applying a consistent threshold of <1% across subjects to ensure uniformity. Thresholded maps were then binarized to create masks for individual WM tracts. Tract-specific metric values were calculated by overlaying the binary masks on standardized maps for each metric. The mean metric values for each subject and tract were extracted using FSL’s *fslstats* tool, enabling quantitative analysis of WM microstructure. Anatomical identification was done using the Human Xtract atlas.^40^

### Kinarm Task

Our assessment of motor ability used a bimanual BOB coordination task on the KINARM robotic interface because the task provides objective, normalized quantification of bimanual coordination and visuomotor control. The interface displayed a 30 cm virtual bar (1 cm thickness) connecting participants’ index fingers and supported by a 1-cm-radius virtual ball at its center (Figure 1). A gravitational constant (bar mass, 0.166 kg; ball mass, 0.4 kg) created realistic weight cues, and vision of the arms and ball was occluded. Participants were instructed to move the ball “quickly and accurately” to a sequence of 4 circular targets (1 cm radius) arranged 10 cm from the workspace origin, with each target turning from red to yellow once the ball entered and was held for 1 second. The task comprised 3 1-minute levels of increasing difficulty: level 1 fixed the ball to the bar; level 2 allowed the ball to translate along the bar and fall off at ≥ 20° tilt; and level 3 increased the ball’s frictionless rolling speed.

**Figure 1.**
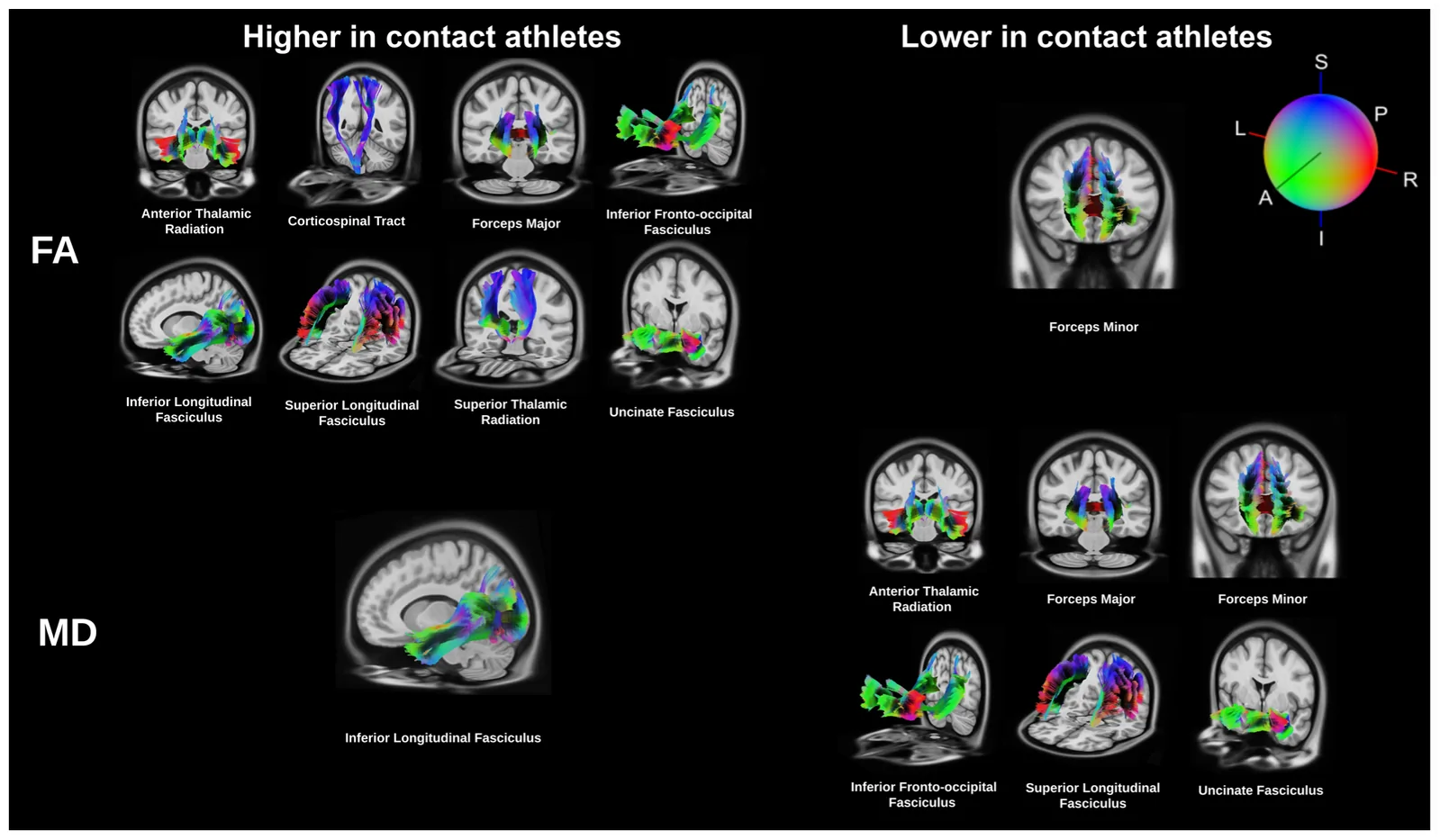
WM tract localization summary for FA (top) and MD (bottom) tracts showing significant preseason between-group differences. Tracts with higher FA or MD in contact athletes relative to noncontact controls are shown on the left, and tracts with lower FA or MD in contact athletes relative to controls are shown on the right. Streamlines are overlaid on the MNI152 template, and color indicates principal diffusion direction according to the orientation sphere. FA, fractional anisotropy; MD, mean diffusivity; WM, white matter; A, anterior; P, posterior; S, superior; I, inferior; L, left; R, right.

### Statistical Analysis

Statistical analyses were determined a priori to test our 3 hypotheses. Data distribution was assessed visually via histograms and Q-Q plots. To examine cross-sectional preseason differences in diffusion metrics between contact athletes and noncontact controls, Welch *t* tests were conducted for each WM tract. *P* values were adjusted using a false discovery rate procedure and statistical significance was set at *P* < 0.05. For longitudinal diffusion analyses, we computed change scores (POST-PRE) for FA and MD in all tracts for HE and LE athletes. We also fitted generalized estimating equation (GEE) models assuming a Gaussian distribution with an identity link and an exchangeable working correlation structure. Participant was specified as the clustering variable, with exposure group and tract included as model factors. Robust standard errors were also used for statistical inference. This approach accounts for correlation among tract-level change scores within the same athlete. All statistical analyses were conducted in R (Version 4.5.1) and Prism (Version 11.0.1).

Behavior on the Kinarm BOB task was quantified using a normalized composite measure of overall task performance, the Task Score.^34,36^ The Task Score combines kinematic metrics such as speed, accuracy, and movement correctness, applying Box-Cox transformations to yield a normalized value where lower scores indicate better performance and higher scores indicate worse performance.^34,36^ Group differences in Task Score change were evaluated using independent *t* tests, with Cohen’s *d* reported as a measure of effect size.

SCAT3 symptom and severity scores were analyzed as change scores (POST-PRE) and compared between groups using Mann-Whitney *U* tests due to skewing. Glass rank-biserial correlation (*r*_rb_) was calculated as the effect size for these Mann-Whitney *U* tests.^38^

## Results

We first examined baseline differences at the preseason timepoint in DTI metrics for each WM tract between contact athletes and noncontact controls (Figure 1). Contact athletes had generally higher FA than noncontact controls in 15 of 16 tracts (all *P* < 0.001; *d* ≥ 2.28; Figure 2a), except for the forceps minor, which was decreased (*P* < 0.003; *d* = –1.13; Figure 2a). For MD, contact athletes showed lower MD in 10 of 16 tracts (*P* ≤ 0.01; *d* ≤ –1.03; Figure 2b), but higher MD in the inferior longitudinal fasciculus bilaterally (ILF_L: *P* < 0.02, *d* = 0.91; ILF_R: *P* < 0.001, *d* = 2.04; Figure 2b). We observed no evidence of baseline MD differences in the corticospinal tract or superior thalamic radiation bilaterally (CST_L: *P* = 0.22, *d* = 0.47; CST_R: *P* = 0.80, *d* = –0.10; STR_L: *P* = 0.17, *d* = –0.50; STR_R: *P* = 0.56, *d* = –0.21; Figure 2b). To test whether head-impact exposure was related to within-season WM change, we compared tract-level FA and MD change scores (POST-PRE) between HE and LE athletes using GEE models that account for within-athlete correlation across tracts. For FA change, the model did not demonstrate a significant exposure-group effect, Wald χ²(1) = 0.14, *P* = 0.71. HE athletes did not differ from LE athletes in FA change, *β* = 0.0016, SE = 0.0042, 95% CI (−0.0067, 0.0098). Similarly, there was no significant exposure-group effect for MD change, Wald χ²(1) < 0.01, *P* > 0.99, with comparable changes observed in HE and LE athletes, β = −0.003 × 10^5^, SE = 1.175 × 10^5^, 95% CI (−2.31 × 10^5^, 2.30 × 10^5^).

**Figure 2.**
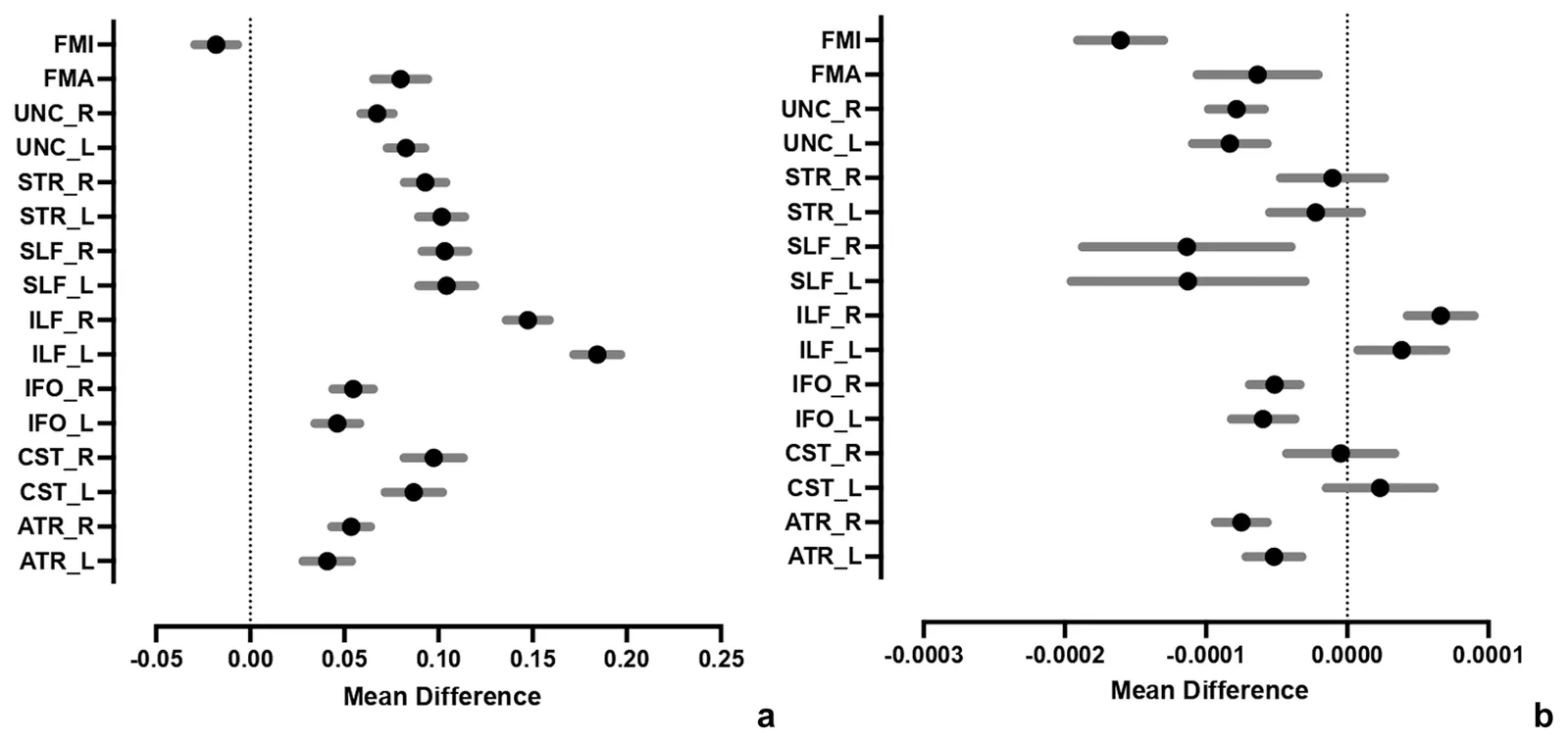
Tract-specific diffusion differences at preseason. (a) FA mean differences between contact athletes and noncontact controls across major commissural and association tracts. (b) MD mean differences for the same tracts. Black circles, mean between-group difference for each tract; gray bars, 95% CI; vertical dotted line, no difference (mean difference = 0). ATR, anterior thalamic radiation; CST, corticospinal tract; FA, fractional anisotropy; FMA, forceps major; FMI, forceps minor; ILF, inferior longitudinal fasciculus; IFO, inferior fronto-occipital fasciculus; _L, left; MD, mean diffusivity; SLF, superior longitudinal fasciculus; STR, superior thalamic radiation; _R, right; UNC, uncinate fasciculus.

Data collected from helmet accelerometers were used to examine the frequency of head impacts over the American Football season for each athlete. The 22 athletes enrolled had total impacts ranging from 33 to 506 based on position (Table 1). As a check after separation into 2 groups based on median impact frequency, we verified that the HE group had significantly more total impacts than the LE group (*P* < 0.001, *d* = 1.71; Table 1). The HE group also experienced significantly higher total *g*-force from these impacts (*P* = 0.001, *d* = 1.71; Table 1). On average, this was 7.20 impacts per session for the LE group versus 14.62 impacts per session for the HE group.

Based on the exposure groups described above, we examined bimanual coordination before and after a single American Football season among athletes with complete pre- and postseason BOB data (HE, n = 5; LE, n = 7). Using the summary Task Score, we tested whether motor ability on this task changed by the end of the season. Athletes in the LE group showed a modest improvement by the end of the season (–0.33 ± 0.25), whereas those in the HE group became worse over time (0.26 ± 0.33), yielding a between-group difference in change of 0.59 (95% CI, 0.22 to 0.96; *P* = 0.005) and a large effect size (*d* = 2.07; Figure 3a).

**Figure 3.**
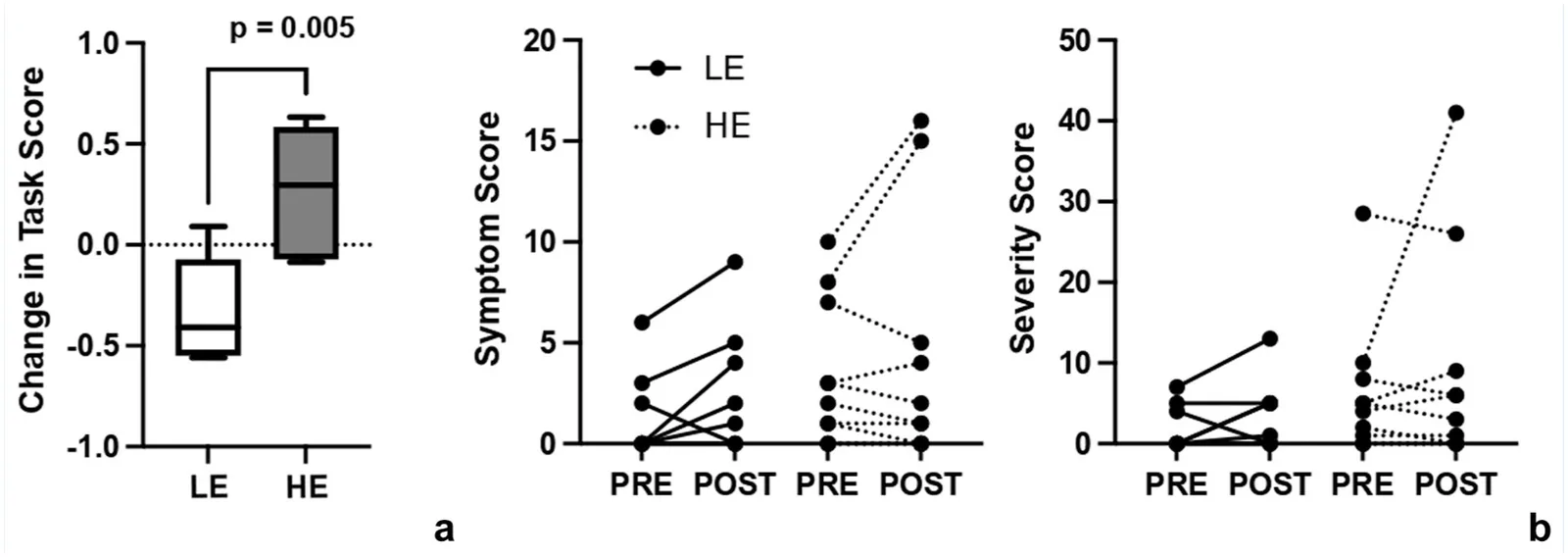
Change in motor task performance and concussion symptoms across the season. (a) Change in Kinarm BOB Task Score from PRE to POST for LE and HE athletes. The *P* value indicates the between-group difference in change. (b) Individual SCAT3 symptom (left) and severity (right) scores at PRE and POST for LE (solid lines) and HE (dashed lines) groups, with lines connecting repeated measures for each athlete. BOB, Ball-on-Bar; HE, high exposure; LE, low exposure; PRE, preseason; POST, 30 days after season end; SCAT3, Sport Concussion Assessment Tool, third edition.

For the SCAT3, baseline scores were low and comparable between groups, with no significant PRE to POST change within either group (Figure 3b). The change in symptom score did not differ between groups (LE, 1.25 ± 1.91; HE, 0.82 ± 2.93; U, 57; *P* = 0.29, *r*_rb_ = 0.30; Figure 3b left). Similarly, the change in severity score did not differ between groups (LE, 1.63 ± 3.42; HE, 2.59 ± 9.62; U = 56; *P* = 0.33, *r*_rb_ = 0.27; Figure 3b right). Symptom and severity distributions showed floor effects, with many athletes reporting zero symptoms and very low severity scores at both time points (Figure 3b). At the individual level, most athletes exhibited only small changes from PRE to POST, with isolated larger increases in severity that did not change the overall between-group interpretation. Overall, these findings indicate no group differences in the trajectories of either symptom or severity scores for HE and LE across the season.

## Discussion

RHI has been implicated increasingly in subtle yet cumulative alterations in WM microstructure, even in the absence of clinically diagnosed concussion.^4,29^ In this pilot study of varsity American football players and noncontact controls, contact athletes began the season with systematic differences in WM microstructure, showing higher FA and predominantly lower MD across most major tracts compared with noncontact controls. Because these comparisons are cross-sectional and occurred at preseason, they may reflect pre-existing factors, previous sport participation, or other unmeasured group differences. Over the course of the season, athletes with higher head-impact exposure showed worsening bimanual coordination on the Kinarm BOB task, whereas LE athletes showed modest improvement. By contrast, SCAT3 symptom and severity scores remained low and did not differ in their trajectories between exposure groups, and GEE models did not detect longitudinal tract-level DTI differences between HE and LE athletes. These findings suggest that objective motor testing may capture functional change in athletes even when symptom scores and tract-averaged DTI show limited within-season change.

### WM Microstructural Differences in Contact Sport Athletes

Extensive imaging evidence indicates that athletes exposed to RHI can show alterations in WM microstructure. Collision-sport athletes often demonstrate higher FA and lower MD in major WM tracts compared with noncontact athletes,^13^ although the opposite pattern has also been found.^5^ DTI differences have been reported in contact-sport athletes even in the absence of diagnosed concussion,^22^ but cross-sectional findings should be interpreted cautiously because they can reflect pre-existing factors and group differences beyond head-impact exposure. Because our contact-control comparisons are cross-sectional, the observed between-group diffusion differences cannot be attributed to the monitored season and may have predated participation in this study. More rigorous matching of controls for factors that can influence diffusion metrics (e.g., athletic background, lifestyle, and socioeconomic status) would improve the interpretability of cross-sectional between-group differences in future studies. Future dose-response analyses should also consider composite risk-weighted impact metrics because this approach may capture biomechanical heterogeneity more sensitively than impact frequency alone.^25^

In the present study, tract-level diffusion changes over 1 monitored season were minimal, and we did not quantify lifetime exposure to RHI. Several methodological and biological factors may contribute to this null longitudinal imaging result over a single season. First, the diffusion acquisition used a single-shell protocol, and, while this is appropriate for tensor estimation, this configuration may have limited sensitivity to subtle microstructural changes expected from months of RHI exposure. Future studies using higher angular resolution and/or multishell diffusion may be better positioned to capture changes beyond standard tensor-derived FA/MD. Second, our analyses used tract-averaged mean FA and MD extracted from tract masks. Head-impact exposure may produce spatially localized alterations (e.g., in a crossing-fiber region or a restricted tract segment) that would be diluted when averaging across the full tract volume. Accordingly, null tract-mean results do not exclude the presence of more focal change. Complementary approaches, such as along-tract or fixel-based analyses, could be used to characterize spatially specific effects. Third, statistical power could have impeded our detection of small longitudinal effects. Our convenience sample and absence of an a priori power analysis mean these results should be used for hypothesis generation and to inform future fully powered longitudinal studies. Finally, the dissociation between motor change and minimal tract-averaged DTI change may reflect differences in sensitivity across measurement domains.

### RHIs and Motor Coordination Deficits

Our results showing declines in bimanual coordination among HE athletes align with an emerging body of work linking RHIs to subtle motor control impairments. Even a single session of subconcussive head impacts has been shown to measurably alter motor system function.^16^ In amateur boxers, sparring without any diagnosed concussions led to increased corticomotor inhibition and a dampening of motor control, evidenced by delayed recruitment of motor units and reduced force output at given activation thresholds.^17^ These findings suggest that subclinical head impacts can subtly alter neuromotor integration and coordination. Behaviorally, athletes with greater head-impact exposure may exhibit slower reaction times, poorer fine motor control, or diminished bimanual task performance, even though such changes might not be obvious without sensitive testing. Although caution is warranted in interpreting these behavioral deficits as task specific or solely caused by the monitored season, the declining BOB performance in HE athletes, contrasted with improvements in LE athletes, supports the possibility that higher head-impact exposure is associated with changes in complex motor task performance.

Consideration of repeat-testing effects is also important for interpreting the BOB task. In healthy young adult athletes, KINARM outcomes show moderate-to-good test-retest reliability for most parameters, indicating that performance is generally stable across sessions when underlying function is unchanged.^30^ A small improvement across repeated testing is therefore plausible and is consistent with the LE group’s modest improvement from PRE to POST. However, familiarization effects would be expected to bias performance toward improvement in both groups; therefore, the HE group’s worsening over the same interval is unlikely to be explained by retesting alone. The BOB task has also been identified as a particularly discriminative task for bimanual coordination deficits in robotic assessment contexts, which supports its sensitivity to subtle bimanual control changes.^15^ These features provide a plausible explanation for why exposure-related motor changes may be detectable over a single season even when tract-averaged diffusion metrics show minimal longitudinal change.

### Symptom-Based Clinical Assessments

Despite clear differences in BOB Task Score change, we found no corresponding changes in SCAT3 symptom and severity scores between HE and LE athletes. Building on Goubran et al,^18^ who found no significant results using the global SCAT score, we examined symptom number and severity scores from the SCAT3 checklist. Scores were low, showed floor effects, and did not differ between exposure groups, suggesting that symptom checklists alone may lack sensitivity to subtle, cumulative effects of RHI over time. Athletes sustaining RHIs may remain asymptomatic, so tools relying on self-reported symptoms or brief clinical screens may not capture objective functional change.^13^ In this study, players in the HE group declined in a complex motor coordination task, yet their symptom checklists remained largely unchanged. These results should not be interpreted as evidence against SCAT3 for its intended acute clinical use; rather, they indicate that the SCAT3 symptom checklist, as used in this historical dataset, was insensitive to the functional change observed in this small cohort.

### Limitations

Several limitations temper interpretation. First, this was a convenience sample without an a priori power analysis, and the Kinarm BOB analysis included only athletes with complete pre- and postseason data, limiting precision and generalizability. Second, controls completed only a baseline MRI. This design means the control group provides a cross-sectional comparison at PRE but cannot address normative seasonal or test-retest diffusion change. The lack of postseason control MRI may also bias interpretation of baseline contact-control differences if unmeasured factors differed between groups, even though controls reported no contact-sport participation. Third, the study did not systematically quantify lifetime concussion burden or other possible sources of RHI. Fourth, the imaging protocol focused on quantitative diffusion MRI and did not include blinded qualitative neuroradiologist review; subtle structural abnormalities outside the DTI-derived metrics may therefore have been missed. Finally, helmet-mounted accelerometers provide useful relative exposure metrics but are vulnerable to measurement noise, and future studies should pair event-level quality control with composite or risk-weighted exposure metrics.

## Conclusion

In this pilot study of 22 contact athletes, American Football players showed preseason WM diffusion differences compared with noncontact controls, but these cross-sectional differences cannot be attributed to the monitored season or to RHI without stronger control matching and lifetime exposure data. Across the season, HE athletes showed worse bimanual coordination than LE athletes, whereas SCAT3 symptom and severity scores remained low and GEE models did not detect longitudinal HE-versus-LE tract-level FA or MD differences. These results should therefore be interpreted as hypothesis generating. Future fully powered longitudinal studies should quantify lifetime concussion and head-impact history, use validated composite exposure metrics, include longitudinal control data and blinded morphologic MRI review, and integrate sensitive behavioral assessment with advanced neuroimaging across multiple seasons.
